# Developing Novel Gas Discharge Emitters of Acoustic Waves in Air for Nondestructive Testing of Materials

**DOI:** 10.3390/s22239056

**Published:** 2022-11-22

**Authors:** Daria A. Derusova, Vitaly O. Nekhoroshev, Victor Y. Shpil’noi, Vladimir P. Vavilov

**Affiliations:** 1 Industrial Tomography Center, Tomsk Polytechnic University, 634050 Tomsk, Russia; 2 Research Lab of Catalytic and Biomedical Technologies, Sevastopol State University, 299053 Sevastopol, Russia; 3 Institute of High Current Electronics SB RAS, 634055 Tomsk, Russia

**Keywords:** gas discharge, laser vibrometry, acoustic wave, nondestructive testing, composite, impact damage

## Abstract

This study was devoted to the development of novel devices and a methodology intended for generating ultrasonic waves in an air medium by using atmospheric pressure gas discharge. In the proposed electrode system, the discharge process was accompanied by the generation of acoustic waves on the emitter surface and, consequently, in the ambient air. The gas discharge emitter vibrations were analyzed by applying the technique of Scanning Laser Doppler Vibrometry (SLDV). It was shown that the magnitude of displacements matched the corresponding characteristics of classical piezoelectric and magnetostrictive transducers. The use of the Fast Fourier transform procedure supplied amplitude–frequency spectra of vibrations generated by the gas discharge emitter. The amplitude–frequency spectrum analysis showed that the proposed emitter was able to generate acoustic waves in the air with frequencies from 50 Hz to 100 kHz, and such a device can be used for the nondestructive testing (NDT) of materials. The results of the statistical analysis of vibration displacements in the repetitive pulsed mode were discussed. A non-stable characteristic of the vibration displacement of the emitter membrane was demonstrated. The parameters of such instability were associated with the features of gas discharge processes. In the experiments, the proposed gas discharge emitter was used in combination with SLDV for inspecting carbon-fiber-reinforced polymer composites. The experiments demonstrated the possibility of using an air-coupled gas discharge transmitter to generate acoustic waves in NDT applications.

## 1. Introduction

The research in the field of air-coupled ultrasonic transducers is a growing trend in various applications of the nondestructive testing (NDT) of composite materials [1,2]. The appearance of air-coupled ultrasonic transducers has led to the development of some novel NDT techniques [3,4] representing a certain alternative to traditional ultrasonic NDT [5,6]. The most important advantage of air-coupled transducers is the absence of added mass and immersion liquids [7].

In conventional ultrasonic NDT, materials are stimulated by means of piezoelectric transducers and, in some particular cases, such as ultrasonic infrared thermography, by magnetostrictive transducers. However, each type of such emitters has certain drawbacks that limits their use in particular test cases [8]. Piezoelectric transducers are limited by the amplitude of applied voltage and, hence, by the emitted power. In addition, they are fragile and characterized by a relatively narrow frequency range. Because the resonance frequency of a piezoelectric transducer depends on its size, the higher frequencies require smaller transducers, thus, further limiting stimulation power [9]. In fact, transducer work frequencies and power depend on transducer size and configuration. In turn, magnetostrictive transducers provide a high level of emitted power, but they are strongly limited by their work frequency, thus, becoming inefficient if multicomponent defects are to be evaluated [10,11].

In air-coupled implementations of ultrasonic NDT, the excitation of ultrasonic waves by using a pulsed electric discharge was recently proposed, which produces quick changes of pressure in the ambient environment [12]. An impetuous increase in temperature accompanied by the expansion of the plasma region in the ambient medium produces a compression wave, which can be considered as a shock wave formed as a result of the electro-thermo-acoustic effect. A shock wave propagates in the discharge gap to interact with the electrodes and the discharge camera, thus, causing deviations from their equilibrium position. Correspondingly, during the process of relaxation, elastic waves are generated in a wide frequency range. The energy of vibrations is partially transformed in the ambient environment to form acoustic waves [13]. However, the mechanism described is scarcely studied, and it is believed that the analysis of the operation of air-coupled (non-contact) transducers represents an actual topic for further research.

Starting from the 1980s, a couple of publications devoted to the excitation of acoustic vibrations by open plasma spark sources have appeared. For instance, it was shown in [14] that a high-voltage source of spark discharge can produce in the air a high-pressure pulse accompanied by rapidly growing air pressure [14]. This leads to the appearance of longitudinal, shear and surface waves. A spark gap device can serve as a source of broadband ultrasonic waves to also be used as a point oscillator. Such sources of ultrasonics are cheaper than pulsed laser devices, and they can be used as common acoustic sources in NDT applications. Some disadvantages of air-coupled devices also were noted, such as possible damage to electrodes and test objects, difficulties in manufacturing high-voltage capacitor banks intended for the excitation of a spark discharge, as well as safety issues. The susceptibility of the above-mentioned devices to electromagnetic interference from the spark discharge was also noted [15].

The investigations of the spark discharge between electrodes placed into a liquid demonstrated the non-linear dependence of the generated acoustic power on cathode–anode voltage [16]. The efficiency of such devices quickly reaches a maximum for low-energy pulses and at shorter distances between the electrodes. With a higher pulse power and longer inter-electrode gaps, the efficiency decreases. The same result was also obtained on a laboratory lightning simulator [17]. Some drawbacks of air-coupled devices were highlighted in [14,15,16,17], namely, the poor directivity of generated acoustic waves and the influence of environmental factors, such as air humidity and the ionization of gas in the inter-electrode space, on the magnitude of amplitude and phase jitter.

The above-mentioned features of plasma spark dischargers prompt the idea of designing a closed chamber where a pulsed discharge takes place, thus, allowing the production of a powerful broadband source of acoustic waves and solving safety operation problems (from physical damage to electromagnetic interference), as well as reducing the dependence of jitter on environmental factors. The proposed emitter design allows directing the acoustic wave front parallel to the membrane surface. In addition, by properly choosing the membrane material and thickness, one can define the required frequency operation range of the emitter. Generally, the implementation of the above-mentioned features can make the proposed air-couple emitters useful for NDT purposes.

In this study, a novel type of gas discharge air-coupled ultrasonic emitters is proposed, and its vibration characteristics are investigated by using the technique of Scanning Laser Doppler Vibrometry (SLDV). Moreover, this technique is an effective tool for analyzing vibrations in solid objects, thereby allowing one to evaluate the directivity of generated acoustic waves and visualize their spatial distribution [18,19,20].

## 2. Measurement of Vibration Characteristics

### 2.1. Experimental Setup

**Figure 1:**
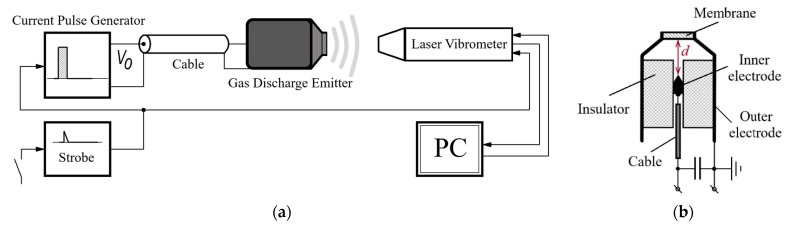
Simplified functional diagram (**a**) of the setup used for investigation of vibration characteristics of a gas discharge emitter (**b**).

Air-coupled gas discharge emitters represent a novel type of non-contact devices, which are able to generate acoustic waves in a pulsed mode. Their operation principle is based on using the gas pressure rise in a gas discharge gap, which appears under the warming up and expanding of the discharge plasma area due to current pulse flow. The pressure jump, which forms in the inter-electrode space, unbalances the emitter membrane, thus, leading to the generation of acoustic waves in the ambient air. The technical performance of a gas discharge emitter was investigated by using the SLDV setup, as shown in Figure 1.

The experimental setup includes a gas discharge emitter of acoustic waves, communication line and current pulse generator, as well as a scanning laser Doppler vibrometer PSV-500-3D-HV (Polytec, Germany), as shown in Figure 1. The discharge was excited by using a home-made current pulse generator with an output voltage of V_0_ < 12 kV and short-circuit current up to1 kA. In the experiments, the pulse repetition rate varied up to 3 Hz in order to avoid possible overheating of the electrodes during multiple measurements and the necessity to ventilate the discharge chamber. It is worth noting that the chamber design allowed us to reach a pulse repetition rate of 60 Hz and higher. The upper repetition rate limit was 500 Hz, which corresponded to the continuous operation mode of the emitter. However, this was beyond the scope of this research. The inter-electrode gap (specified as *d* in the Figure 1b) was 5 mm. The Fast Fourier Transform (FFT) spectrometer option with a resolution of 30 Hz was used to analyze the amplitude–frequency spectrum of generated vibrations.

### 2.2. Modeling

The resonance frequencies of the emitter depend on the size and properties of the membrane; however, the discharge itself appears broadband [14,15,16,17]. Therefore, the development of a membrane model simulating the vibrational characteristics of objects seems to be appropriate in order to minimize the size of experiments and correct some object properties, such as the mass/size ratio and rigidity and/or damping. The analysis of the dynamic characteristics of objects, such as the vibration parameters (mode shape and frequency), is useful for altering object resonance frequencies.

The FEM modeling of vibrations in the emitter membrane was based on Basic Modal Analysis by using Autodesk Inventor software. The model included solid-type volume elements with properties similar to a tested material, in particular, aluminum, see [21] for details.

In general, the calculation of membrane natural frequencies requires the knowledge of its physical properties, which are united under the term of bending stiffness (*D*) [22]:

(1) $$D=\frac{Eh^{3}}{12\left(1-v^{2}\right)}$$

where *h* is the membrane thickness, *E* and *v* are the Young modulus and Poisson ratio of the material, respectively.

The natural frequencies depend on the sample geometry and on the support conditions on the edges. In this study, a disk-like aluminum membrane with a clamped edge support was considered. In the classical approach, the fundamental resonance frequency of a circular membrane with a clamped edge was recently suggested in [22]:

(2) $$\omega_{0}=\sqrt{\frac{192\pi D}{1.8mr^{2}}}$$

where *m* is the mass of the membrane and *r* is the radius.

For instance, by substituting Equation (1) into Equation (2) with *ω* = 2*πf*, one obtains [22]:

(3) $$f_{0}=\frac{10.3h}{R^{2}}\sqrt{\frac{E}{12\left(1-\nu^{2}\right)\rho }},$$

where ρ is the material density.

**Figure 2:**
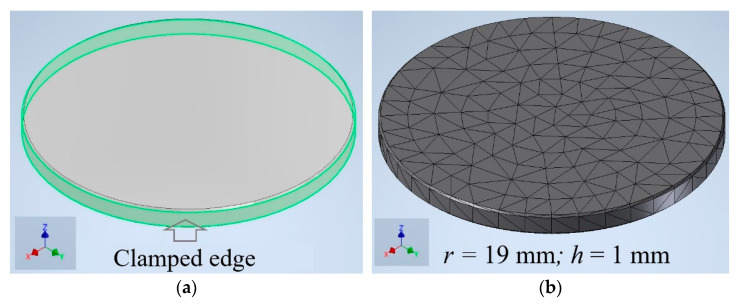
Aluminum membrane: finite element model (**a**) and numerical mesh (**b**).

The resonance frequency of the emitter membrane was calculated for the following experimental parameters: *r* = 19 mm and *h* = 1 mm. The aluminum mechanical properties were: *E* = 70 × 10^9^ Pa, *v* = 0.34 and ρ = 2700 kg/m^3^. Figure 2 shows the used finite element model (Figure 2a) and the respective numerical mesh (Figure 2b) with a mean element size of 0.3 mm; the computation time was 10 s.

The results of the calculation were of the membrane resonance frequency *f_m_* and distributions of mechanical vibrations by *z* coordinates. In the model, the boundary condition involved the clamped edge support, thus, well correlating the real experiment. Additionally, Basic Modal Analysis assumed that the model was dynamically tested in ideal conditions, i.e., without taking into account additional internal and external factors. The experimental values of the resonance frequency *f_r_* determined by SLDV, given below, are to be used for validating the mathematical model.

### 2.3. Analyzing Membrane Natural Frequencies: Experimental and Modeling Results

The gas discharge process in the electrode system of the emitter led to the appearance of acoustic vibrations on the emitter surface and, correspondingly, in the ambient air. By using the SLDV technique in time mode, the damped vibrations of the emitter membrane were measured during discharge current pulse flow. A typical graph of damped vibrations in the center of the 1 mm thick aluminum membrane is shown in Figure 2.

**Figure 3:**
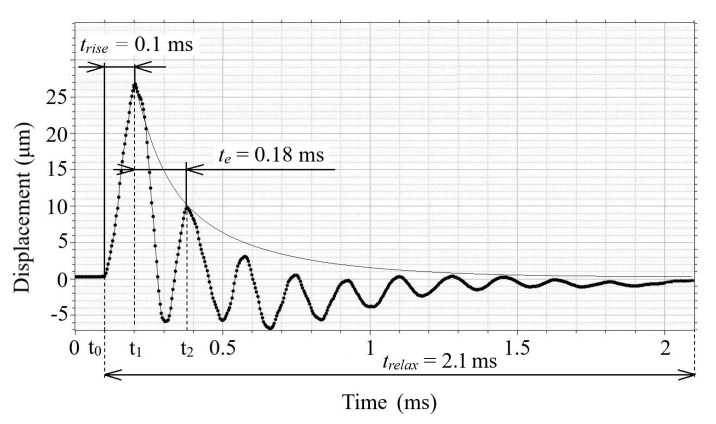
Vibration profile of 1 mm thick aluminum emitter membrane excited by gas discharge electric pulse.

We considered the oscillations of the gas discharge emitter membrane excited by an electric current pulse. It follows from the graph in Figure 3, wherein the amplitude of the first maximum of membrane oscillations $x\left(t_{1}\right)$ reached 26.7 μm at 0.1 ms. The second maximum $x\left(t_{2}\right)$ of the damped oscillations (at 9.9 μm) appeared at the time $t_{e}$ = 0.18 ms when the oscillation amplitude decayed by *e* times in respect to the maximum value. In turn, the time of full signal relaxation was 2.1 ms. In general, the results above show that when the grade of damping of the oscillating system is low, it can be corrected by changing damping characteristics in particular applications.

**Figure 4:**
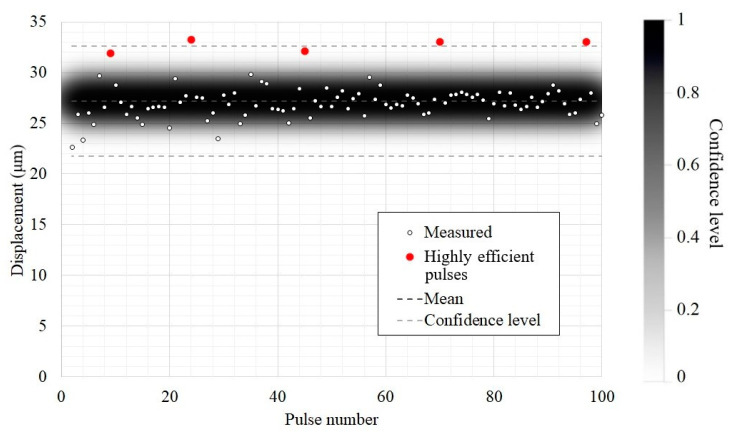
The displacement amplitude in the membrane center measured for 100 pulses (the scale on the right level of confidence).

The magnitude of displacements during the flow of the electric current was evaluated in 100 pulses. All measurements were conducted at a pulse repetition rate of 2.5 Hz. Figure 4 shows these displacements in the center of the membrane.

The analysis of obtained results shows that the amplitude of vibration displacements at the end of the emitter varied from 22.7 to 33 μm. It can be seen that only 3 measurements out of a total of 100 fell out of the confidence level interval, thus, corresponding to 95% confidence. In those three measurements, the signal amplitudes exceeded 33 μm, and, in two other measurements, they reached 32 μm, thus, still being within the confidence level interval. Such five signal peaks appeared once every 20 measurements, and this corresponded to the most efficient regime of acoustic wave generation under the conditions described. It was probably related to both variations in the discharge parameters and physical processes in the gas discharge gap. The mean displacement amplitude was 27.2 μm, thus, matching well with the corresponding characteristic of classical piezoelectric and magnetostrictive transducers [23].

A recent study demonstrated that an air-coupled magnetostrictive transducer (resonance frequency 22 ± 1.65 kHz), in combination with high-power ultrasonic generator (electrical power 0.63 ± 10% kW), provides a vibration displacement at the horn end of up to 30 μm by optimizing the horn design [13]. Commercial piezoelectric transducers, for example, Ultran ACU (resonance frequency 110 kHz), have revealed a vibration displacement of about 10 μm at the emitter end (generator voltage 70 V and alternating current frequency 113 kHz) [13]. In the same study [13], it was shown that continuous ultrasonic excitation using both piezoelectric and magnetostrictive transducers is accompanied by distortions in the amplitude of the resulting signal because of interference between incident and reflected waves. The distortion magnitude depends on the distance between the emitter and a test object. Therefore, in order to achieve optimal matching of the entire acoustic system, the air gap thickness should be multiple to the half-length of the acoustic wave propagating in the air. This operational problem of non-contact acoustic devices can be solved by applying broadband pulsed stimulation implemented in gas discharge devices.

**Figure 5:**
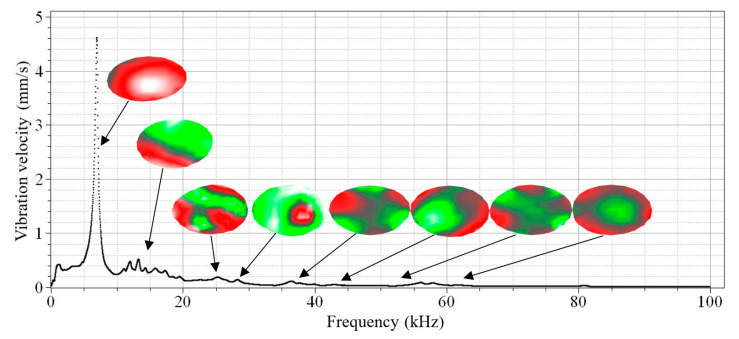
Amplitude–frequency vibration spectrum in the center of aluminum membrane at frequency range from 50 Hz to 100 kHz; 3400 lines; and 30 Hz frequency resolution.

To continue the testing of the oscillations of the gas discharge emitter membrane, the vibration signal was converted into amplitude spectrum in order to demonstrate characteristic frequency domains (Figure 5).

As follows from Figure 5, the amplitude of vibration velocity is smooth through the entire frequency spectrum with a central resonance peak appearing at 7 kHz that corresponds to the membrane eigenfrequency. There is no contact between the plasma and the ambient air in this configuration, and this might be important in practical applications. However, in this case, the amplitude–frequency characteristic of the system will be determined by the membrane rather than the discharge parameters. In particular, the velocity amplitude reaches 4.62 mm/s with a high quality factor (*Q* ≈ 15), and this may further be used for compensation purposes by introducing damping elements. Generally, an atmospheric pressure gas discharge can be used to generate acoustic waves in the range from 50 Hz to 100 kHz and, thus, perform the acoustic stimulation of composite materials.

Both analytical and numerical modeling can also be helpful for explaining some spectral resonances. The Autodesk Inventor software was used to visualize the eigenmodes of vibrations and compare the experimental and theoretical results of the membrane by applying finite element analysis. In the model, the membrane was fixed by the perimeter to simulate real conditions where the sample was fixed by a clamping flange. The modeling allowed us to determine 20 resonance modes in the range from 0 to 100 kHz and compare them with the experimental values of the electric pulse emitter resonances ($f_{max}$) obtained at frequencies from 10 Hz to 100 kHz (Table 1). In addition, these data were used to explain the origin of the resonance peaks in the amplitude–frequency spectrum of the gas discharge emitter. Table 1 and Figure 5 present the results of vibration modeling in the aluminum membrane with clamped edges, along with the estimates of relative measurement errors $\varepsilon \;=\;\left(f_{m}\;-\;f_{r}\right)/f_{r}$, where $\;f_{m}$ and $f_{r}$ are the modeled and experimental resonance frequencies (the experimental data were obtained by using the laser vibrometry technique).

The results above (Table 1 and Figure 5) demonstrate that, with increasing frequencies, one observes the decrease in both vibration velocity amplitudes on the emitter surface, as well as the decrease in their root-mean-square values. In the frequency range analyzed, the maximum amplitude of vibration velocity was 4.62 mm/s at a frequency of 6.69 kHz. The minimum velocity amplitude of 0.006 mm/s appeared at 84 kHz, while the mean value was 0.132 mm/s. This proves that the main energy of emitter surface vibrations is concentrated in a frequency range of up to 10 kHz, even if some peaks were observed at frequencies higher than 80 kHz.

It is worth noting that the discrepancy between the theoretical and experimental values of resonance frequencies did not exceed 5%. The spatial distribution of vibrations, which was evaluated by means of laser vibrometry, matched well with the results of modal analysis, and some disturbances were caused by non-stationary phenomena in the plasma, as well as, probably, by uneven clamping of the membrane. In addition, the resonance frequency of the discharge emitter electrode significantly influences the work frequencies of the emitter, which was confirmed by the analysis of amplitude–frequency spectrum. Hence, the use of different types of electrodes may help to produce the required resonance frequencies of electrodes and, thus, define the acoustic spectrum of gas discharge systems.

### 2.4. Spectral Characteristics of Metal Membranes

**Figure 6:**
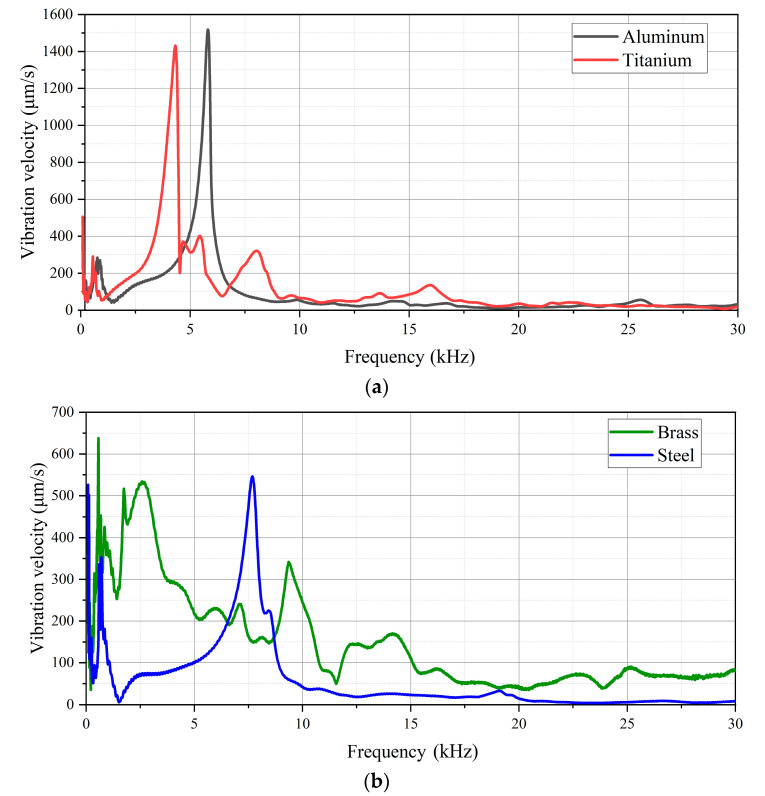
Amplitude–frequency spectrum of 2 mm thick metallic membranes (aluminum and titanium (**a**); brass and steel (**b**)).

In order to analyze the peculiarities of the operation of gas discharge emitters and optimize their configuration, some membranes made of different metals were investigated. In particular, these metals were: aluminum (D16T), brass (LS59), titanium (BT6-1) and steel (St3). Each sample series included five membranes with a size of 38 × 2, 38 × 2.3 and 38 × 2.6 mm. Figure 6 shows the amplitude–frequency spectrum of vibrations in a frequency range from 1.5 kHz to 30 kHz for four types of metals.

As shown in Figure 6, different membranes reveal different amplitude–frequency spectra even in the case of membranes of the same size and thickness. In the case of Al, the resonance frequency was 5.81 kHz, the resonance frequency of titanium was 4.32 kHz and the resonance frequency of steel was 7.68 kHz. The resonance peaks in the brass membrane were less obvious compared to other materials, i.e., in this case, the amplitude–frequency spectrum was smoothed with a maximum at 2.57 kHz. Oppositely, the aluminum and titanium membranes were characterized by pronounced resonance peaks. In these cases, the vibration amplitudes (aluminum 20.3 μm and titanium 20.1 μm) were higher than for other metals. In steel membranes, one may also observe a characteristic resonance maximum with an amplitude of vibration velocity similar to that in a brass membrane but 2.5 times lower compared to aluminum and titanium. The vibration peculiarities above can be explained by different physical properties of the metals that affect the work frequencies of membranes. It is also worth noting that the presence of high-frequency peaks in the spectra investigated takes place because of the activation of membrane higher resonance harmonics during their relaxation.

Notably, the vibration energy is mainly emitted in the 1.5–11 kHz frequency range, where spectral maximums for all investigated metals are located. However, brass membranes should be recommended for NDT purposes due to their smoother amplitude–frequency spectrum in regard to other metals.

**Figure 7:**
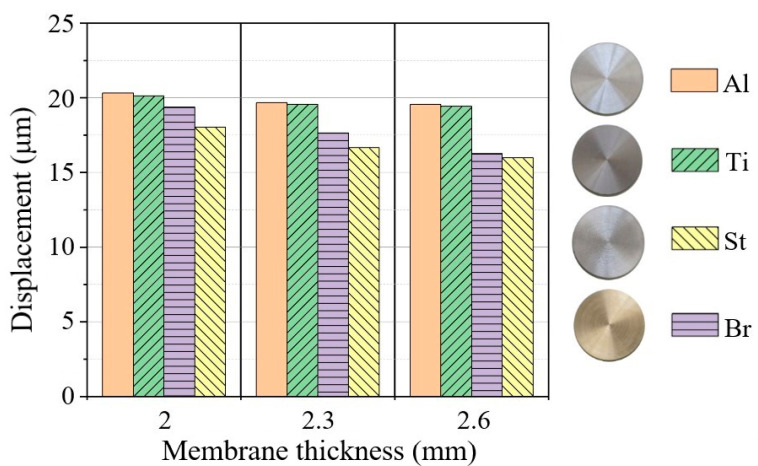
Vibration displacement amplitude vs. membrane thickness (aluminum, titanium, brass and steel membranes).

The data above prompts that the desired frequency range of emitters can be defined by optimizing the material, size and geometry of membranes according to practical requirements. Figure 7 shows histograms of vibration displacements for metallic membranes of varying thickness. It can be seen that displacement amplitudes are different for different metals and slightly decrease for thicker membranes, provided the electrical power of the generator is constant. In the center of aluminum and titanium membranes, the maximum displacement amplitude reached 20 μm. In these membranes, both the frequency and amplitude of vibrations are related to the lower density of the metals when compared to steel and brass.

**Figure 8:**
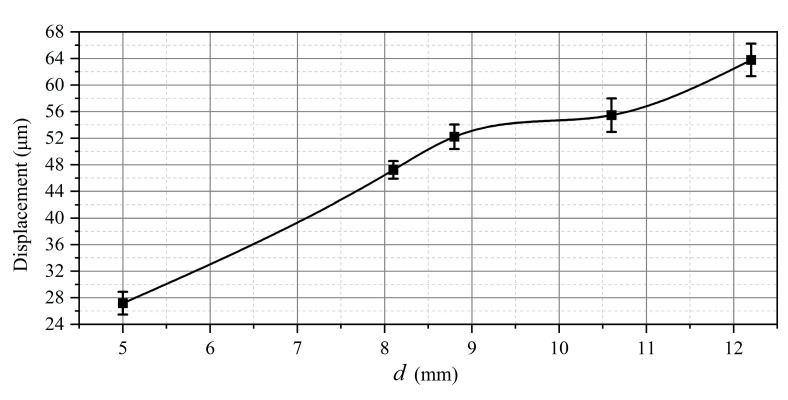
Vibration displacement amplitude vs. inter-electrode gap thickness (membrane thickness 1 mm).

The influence of the inter-electrode distance on displacement amplitude was investigated on the aluminum membrane (diameter *D* = 38 mm and thickness *h* = 1 mm). The inter-electrode distance was varied by changing the position of the potential electrode with respect to the membrane. The minimal gap thickness was 5 mm, the channel diameter was 3 mm, and the gas discharge volume varied approximately from 15 to 35 mm^3^. In all cases, the pulse repetition rate was 2 Hz, and the variation of the vibration displacement in time was measured by means of laser vibrometer. The corresponding graph is presented in Figure 8 for the aluminum membrane and different inter-electrode gap thicknesses.

The electric field strength, which is necessary for breakdown, was practically constant. With thicker gaps, the breakdown voltage increases, while the energy stored in the capacitor is proportional to the square of the voltage. On the other hand, by assuming the membrane rigidity constant, the energy at the point of maximum displacement will be proportional the square of the displacement. This would lead to the direct proportion between the displacement and gap thickness *d*. However, one more factor is the gap volume, which acts as a kind of a muffler. Moreover, discharge resistance may increase with longer discharge gaps, thus, decreasing the current in the discharge contour. Therefore, the increase in *d* by 50% leads to an increase in the displacement amplitude by only 40%.

## 3. Nondestructive Testing of Polymer Composites: Experimental Results

**Figure 9:**
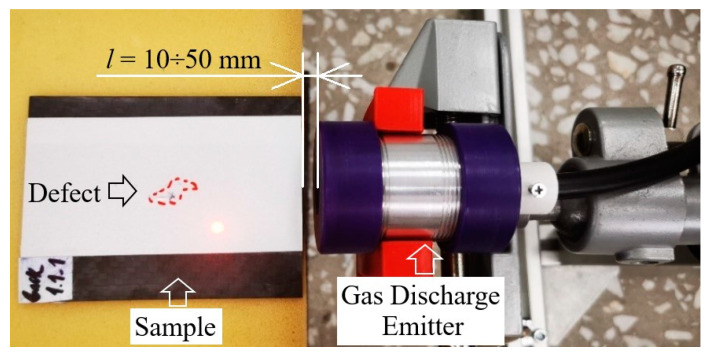
Acoustic stimulation of CFRP composite with 15 J impact damage by using air-coupled gas discharge device.

The use of air-coupled ultrasonic excitation allowed us to expand the potentials of composite inspection, simplify tests and minimize external impacts on test objects compared with traditional NDT techniques. In this study, some experiments on the detection of impact damage in CFRP were conducted by using a new type of air-coupled transducer. A gas discharge emitter was used to evaluate a 15 J impact damage in a 100 × 75 × 1.1 mm^3^ CFRP sample with the distance between the sample and the emitter varying from 10 mm to 50 mm, as seen in Figure 9. The CFRP physical properties were: conductivity 0.64 W × m^−1^ × K^−1^, diffusivity 3.82 × 10^−7^ m^2^ × s^−1^, density 1560 kg × m^−3^ and sound velocity 1133 m × s^−1^ [24]. The CFRP laminate configuration was [90/0/−90]_s_, and the overall fiber volume fraction was equal to 0.60. The size of the impact damage area was approximately determined, by means of infrared thermography, to be 7.1 × 18.2 mm^2^.

By performing SLDV in the Fast Fourier transform mode, vibration spectra were measured on the sample surface at each measurement point. In a frequency range from 50 Hz to 100 kHz, 3200 spectral lines with a step of 31.25 Hz were defined. The scan mesh step was 2 mm, thereby resulting in a total scan time of about 20 min for the above-mentioned frequency resolution. In fact, the discharge pulse rate can be increased to reduce the experiment duration, but the temperature of the emitter should be carefully controlled in this case.

**Figure 10:**
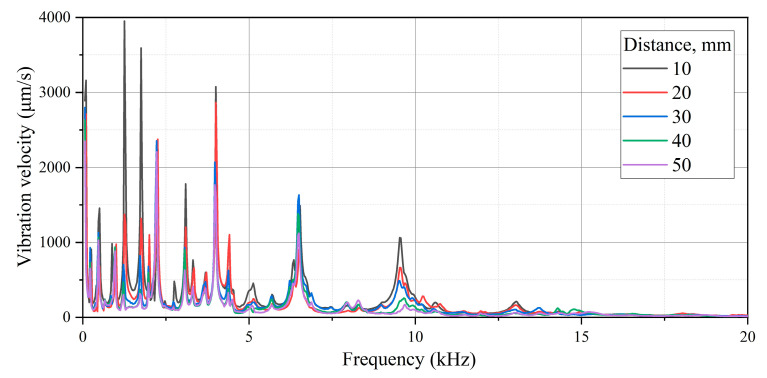
Amplitude–frequency spectra of vibrations in CFRP composite (frequency range from 50 Hz to 20 kHz, emitter–sample distance varies from 10 to 50 mm).

Because a short discharge pulse contains multiple frequencies, the plate under investigation will oscillate under all frequencies, which are present in the pulse. However, it is worth noting that the non-resonance frequencies will quickly decay, thus, underlying object eigenfrequencies. Figure 10 shows the scaled vibration spectrum in the range from 50 Hz to 20 kHz with a number of resonance peaks at the amplitude–frequency spectra of vibrations in the composite obtained. The obtained amplitude–frequency spectra show the distribution of vibrations on the surface of the composite while changing the distance between the test object and the membrane. To characterize such spectra, the minimal ($v_{min}$) and maximal ($v_{max}$) vibration velocities at the corresponding frequencies were determined along with the respective mean values across the whole spectrum ($v_{mean}$). Additionally, the apparent lateral dimensions of impact damage (*a* × *b*) were evaluated. The results are presented in Table 2.

By the known velocity of sound in the CFRP composite (c =1133 m/s) and composite density (ρ = 1560  kg/m^2^) [24] and using the determined value of vibration velocity ($v_{mean}$), one can determine the mean acoustic power ($P_{ac}$) carried by acoustic waves through the area (S), which is perpendicular to sound propagation: ${\;P}_{ac}=\rho v_{mean}cS\;$ [25]. The approach above was applied to the acoustic stimulation of the CFRP composite by means of a gas discharge emitter with the object emitter distance changing from 10 to 50 mm (Table 2).

The data in Table 2 show that, on the sample surface, the vibration amplitude decayed with an increasing distance between the sample and the emitter. This led to a decrease in the acoustic energy effectively pumped into the composite because of the damping of acoustic waves in the air. In parallel, this led to the smaller apparent lateral size of the defect, thus, worsening the efficiency of NDT in general.

**Figure 11:**
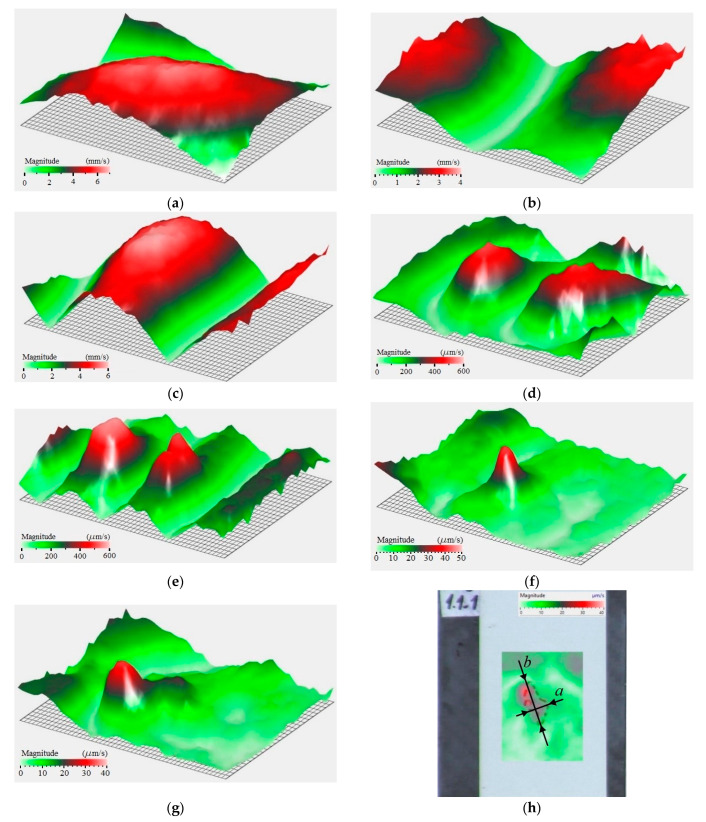
Characterizing impact damage in 1.1 mm thick CFRP sample under broadband excitation: (**a**) 1.75 kHz; (**b**) 2.25 kHz; (**c**) 4 kHz; (**d**) 10.6 kHz; (**e**) 13 kHz; (**f**) 27.2 kHz; and (**g**,**h**) 28.9 kHz.

Figure 10 shows the presence of characteristic resonance peaks in the spectrum, which are conditioned by both natural resonances of the CFRP sample and some local resonances appearing in the zone of impact damage. Some particular vibrograms reflecting the distribution of vibrations at selected frequencies are presented in Figure 11 while using the gas discharge emitter. The images in Figure 11a–e show eigenfrequencies of the CFRP sample, which correspond to the resonance peaks in the 2.25–13 kHz range, as seen in Figure 10. Despite low vibration signal amplitudes in the 15–100 kHz frequency range, the high sensitivity of laser vibrometry allows one to investigate vibrations across the whole spectrum. For example, in the range from 23.9 to 30 kHz, the resonance phenomena were identified in some sub-areas (Figure 11f), as well as across the entire area of impact damage (Figure 11g,h). In general, the obtained results demonstrated a reasonable efficiency of using gas discharge emitters for the acoustic stimulation of composites containing area impact damage defects in the 0.05–100 kHz frequency range, even if the acoustic energy was about 0.2 ÷ 0.75 μW.

## 4. Conclusions

This study was devoted to the development of gas discharge ultrasonic emitters, which enabled generating acoustic waves in the air. The use of a pulsed electric discharge allowed us to develop a powerful, broadband acoustic source for NDT purposes, thus, suggesting a new solution in the field of air-coupled ultrasonics.

In the proposed device, the configuration of the electrodes placed in the isolated chamber prevents the ejection of plasma into the ambient environment. Such a solution is of practical interest because test objects are protected from damage, and the device provides no electromagnetic interference while still transmitting ultrasound in a non-contact way. It is important that one of the electrodes is made as a disk, thus, playing the role of the emitting membrane. In this case, the properties of the membrane define the amplitude–frequency characteristic of the device. Thus, the emitter frequency range can be optimized by properly choosing the material and thickness of the membrane.

The statements above were experimentally confirmed by investigating the vibration characteristics of a gas discharge emitter by using Scanning Laser Doppler Vibrometry. The relationships between emitter work frequencies and membrane type and size were investigated. The vibration velocity and displacement amplitude measured in the membrane center increased in the case of thinner membranes but were strongly affected by the types of metals. In fact, the type of membrane metal determined both the principle membrane resonance and the composition of the work frequency spectrum. This allowed optimizing the parameters of acoustic stimulation in practical applications. In particular, it was experimentally shown that the mean displacement of the 1 mm thick aluminum membrane was about 30 μm at a charging voltage of 12 kV, and, by increasing the pulse energy, this value may exceed 60 μm. It was shown that the displacement magnitude was approximately linear-dependent on the energy stored in the capacitor by the time of discharge. Therefore, by changing the inter-electrode space, one may change the power of ultrasonic stimulation.

The mean displacement amplitude of the membrane in the gas discharge emitter matched well with the corresponding characteristic of classical piezoelectric and magnetostrictive transducers, but an essential advantage of air-coupled transducers is their broad frequency band. Because a short pulse contains multiple frequencies, there is no need in the phase matching between incident and reflected waves in the space between the emitter and a test object. In turn, the interference of waves is characteristic of classical air-coupled devices of continuous operation, including piezoelectric and magnetostrictive. Poor matching of vibration phases in the standing wave appearing between the emitter and a test object may lead to the distortion of the resulting signal amplitude, thus, significantly affecting the general efficiency of ultrasound transmission.

The practical aspect of this study relates to the investigation of the potential of air-coupled ultrasonic stimulation combined with laser vibrometry in application to NDT. By applying a pulsed gas discharge emitter for the acoustic stimulation of impact damage in the CFRP composite, some selective resonance peaks were observed in the amplitude–frequency spectra. Such peaks appeared due to the natural vibrations of the test sample, as well as local resonances over the damaged areas of the composite. The power of acoustic waves generated at distance from 10 to 50 mm was from 0.2 to 0.75 μW, which was high enough for the reliable identification of impact damage in CFRP.

It is worth noting that, in this study, the pulse repetition rate was under 3 Hz in order to diminish the possible overheating of electrodes during multiple measurements and to avoid the necessity to ventilate the discharge chamber, as well as slow down electrode corrosion. Presently, the proposed chamber design allowed us to reach a pulse repetition rate of 60 Hz and higher. The upper repetition rate limit was 500 Hz, thus, corresponding to the continuous operation mode of the emitter. A more detailed analysis of this issue was beyond the scope of this study but may be an interesting topic for further research.

## Figures and Tables

**Table 1:** Modeling results and experimental data on spectral characteristics of electric pulse emitter.

Frequency range, kHz	5.2–8.7	15–16.2	24.4–26	28–28.6	35.8–37	42.5–43.4	49.2–50.2	57–58.9
*v_max_*, mm/s	4.6	0.35	0.19	0.144	0.106	0.051	0.033	0.08
*f_r_*, kHz	7.0	15.8	25.4	28.3	36.5	43.0	49.5	57.9
*f_m_*, kHz	7.2	15.1	25.0	28.1	36.7	43.3	50.2	60.5
*ε*, %	3.3	5	1.6	0.7	<1	<1	<1	4
Resonance modes (experimental)	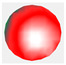	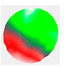	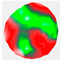	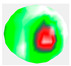	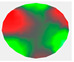	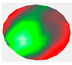	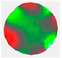	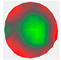
Resonance modes (modeling)	** 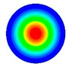 **	** 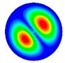 **	** 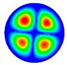 **	** 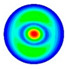 **	** 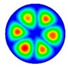 **	** 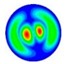 **	** 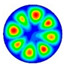 **	** 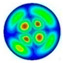 **

**Table 2:** Amplitude–frequency characteristics of vibrations in CFRP composite at different distances between emitter and sample (sample size 100 × 75 × 1.1 mm^3^ and impact damage 15 J).

*l*, mm	*v_min_*, mm/s	*v_max_*, mm/s	*v_mean_*, mm/s	*a × b*, mm	*P_ac_*, μW
10	0.005	4.55	0.101	6.8 × 17.8	0.75
20	0.005	3.95	0.09	5.7 × 10.4	0.6
30	0.005	2.44	0.072	5.7 × 4.4	0.4
40	0.004	2.09	0.056	5.5 × 9.8	0.25
50	0.004	1.92	0.051	2.7 × 3.1	0.2
